# Disruption in the regulation of casein kinase 2 in circadian rhythm leads to pathological states: cancer, diabetes and neurodegenerative disorders

**DOI:** 10.3389/fnmol.2023.1217992

**Published:** 2023-07-05

**Authors:** Md. Zubbair Malik, Mohammed Dashti, Yasmin Fatima, Arshad Channanath, Sumi Elsa John, R. K. Brojen Singh, Fahd Al-Mulla, Thangavel Alphonse Thanaraj

**Affiliations:** ^1^Department of Genetics and Bioinformatics, Dasman Diabetes Institute, Kuwait City, Kuwait; ^2^Department of Computational Biology and Bioinformatics, Sam Higginbottom Institute of Agriculture, Technology and Sciences (Formerly Allahabad Agricultural Institute-Deemed University), Allahabad, India; ^3^School of Computational and Integrative Sciences, Jawaharlal Nehru University, New Delhi, India

**Keywords:** circadian rhythm, PER protein, molecular noise, cellular state, mathematical modeling, pathological states

## Abstract

**Introduction:**

Circadian rhythm maintains the sleep–wake cycle in biological systems. Various biological activities are regulated and modulated by the circadian rhythm, disruption of which can result in onset of diseases. Robust rhythms of phosphorylation profiles and abundances of PERIOD (PER) proteins are thought to be the master keys that drive circadian clock functions. The role of casein kinase 2 (CK2) in circadian rhythm *via* its direct interactions with the PER protein has been extensively studied; however, the exact mechanism by which it affects circadian rhythms at the molecular level is not known.

**Methods:**

Here, we propose an extended circadian rhythm model in Drosophila that incorporates the crosstalk between the PER protein and CK2. We studied the regulatory role of CK2 in the dynamics of PER proteins involved in circadian rhythm using the stochastic simulation algorithm.

**Results:**

We observed that variations in the concentration of CK2 in the circadian rhythm model modulates the PER protein dynamics at different cellular states, namely, active, weakly active, and rhythmic death. These oscillatory states may correspond to distinct pathological cellular states of the living system. We find molecular noise at the expression level of CK2 to switch normal circadian rhythm to any of the three above-mentioned circadian oscillatory states. Our results suggest that the concentration levels of CK2 in the system has a strong impact on its dynamics, which is reflected in the time evolution of PER protein.

**Discussion:**

We believe that our findings can contribute towards understanding the molecular mechanisms of circadian dysregulation in pathways driven by the PER mutant genes and their pathological states, including cancer, obesity, diabetes, neurodegenerative disorders, and socio-psychological disease.

## Introduction

Life is a manifestation of rhythms. One such rhythm is circadian, an endogenous biological process with an oscillation of an approximately 24-h cycle (Dalchau et al., 2018; Lane et al., 2023). The circadian rhythm is present in a wide range of living systems, such as plants, animals, fungi, and cyanobacteria (Goldbeter, 2002; Gonze et al., 2002; Albrecht, 2010; Lane et al., 2023). Although these rhythms are self-sustained and endogenous, they can be entrained to the local environment by external factors (zeitgebers), such as daylight, temperature, and molecular fluctuations (Zeng et al., 1996; Yu and Hardin, 2006). These environmental and metabolic stimuli (e.g., dietary intake) regulate biological processes, such as the sleep–wake cycle, energy metabolism, hormone and immunological responses, and cell proliferation (Koronowski, 2021; Lee, 2021).

A panoply of researchers has reported that changes in the rhythmic process may lead to different diseases (Zeng et al., 1996; Foster et al., 2013; Tan et al., 2018; Mokhlesi et al., 2019). Onset of diseases, such as mood and sleep disorders, cancer, obesity, and diabetes, are all strongly linked with disturbances in these rhythms caused by factors, such as chronic jet lag, eating late at night, sleep deprivation, variations in sunlight, and modifications in hormone regulation (including progesterone and testosterone) (Amaral et al., 2014; Lee, 2021). The expression and activity of various oncogenes and tumor suppressors, in both tumor tissues and the host, are extensively altered by environmental and genetic disruptions in circadian rhythms. Such alterations lead to the incidence and progression of cancer (Papagiannakopoulos et al., 2016; Lee et al., 2019). Circadian disturbances can influence the immunological and metabolic functions of the host, favoring permissive tumor microenvironments in different types of cancer (Aiello et al., 2020; Hadadi et al., 2020). It is further known that disruption of the molecular clock in skeletal muscle promotes insulin resistance and obesity (Dyar et al., 2014). Although it is known that disrupted circadian rhythms affect metabolism, its impact on patients with type 2 diabetes (T2D) is not well-understood.

Circadian rhythm is caused by a genetic regulatory negative feedback loop that involves several clock genes and proteins in the biochemical reaction model (Gerstner and Yin, 2010; Duong et al., 2011). An essential enzyme known to be at the heart of self-sustaining circadian clocks in fungi, plants, and animals is casein kinase 2 (CK2) (Akten et al., 2003; Filhol and Cochet, 2009). CK2 is a ubiquitous eukaryotic protein kinase present in both the nucleus and cytoplasm (Litchfield, 2003; Tsuchiya et al., 2009). CK2 contributes to a wide variety of physiological functions, complex cellular processes (including DNA repair and cell cycle control), and regulation of cell viability (Lin et al., 2002; Ueda et al., 2005). It destabilizes and phosphorylates the TIMELESS (TIM) and PERIOD (PER) proteins in Drosophila, which subsequently suppress the transcriptional activity of the *CLOCK* (*CLK*) gene (Mizoguchi et al., 2006; Szabó et al., 2013). The CLK activator is directly targeted by CK2. It is commonly found in tetrameric complexes formed from catalytic subunits (α and/or α’) and two regulatory β subunits and is traditionally categorized as a messenger-independent protein threonine/serine kinase (Lu et al., 2011; Lee and Kim, 2014). Furthermore, CK2 phosphorylates TIM and PER *in vitro*, indicating that it has an impact on these proteins (Edery et al., 1994; Kloss et al., 2001; Miyazaki et al., 2004). It has also been observed that CK2 phosphorylates TIM protein to a lesser extent than PER. These findings support the notion that CK2 directly regulates TIM and PER. Despite the several reports that have been published regarding the role of CK2 in circadian rhythm of Drosophila, the exact mechanism by which it regulates circadian rhythms at the molecular level is still unclear. Moreover, the dynamics of clock proteins modulated by CK2 is still an ongoing research question and needs to be systematically studied.

In the present study, we used a stochastic approach to examine the dynamic behavior of circadian rhythm driven by CK2 and thereby to discern the regulatory role of CK2 at a molecular level. We extended the biochemical pathway model for circadian rhythm in Drosophila by incorporating various possible interactions of CK2 with clock proteins. We present the method to simulate the developed biochemical network model and we discuss the numerical simulation results and interpretations.

## Materials and methods

The extended model of the Drosophila circadian pathway, which incorporates the impact of CK2 and describes the stochastic simulation algorithm used for the simulation of biochemical reaction network, is proposed in the following sub-sections.

### Description of the circadian CK2 model

**Figure 1:**
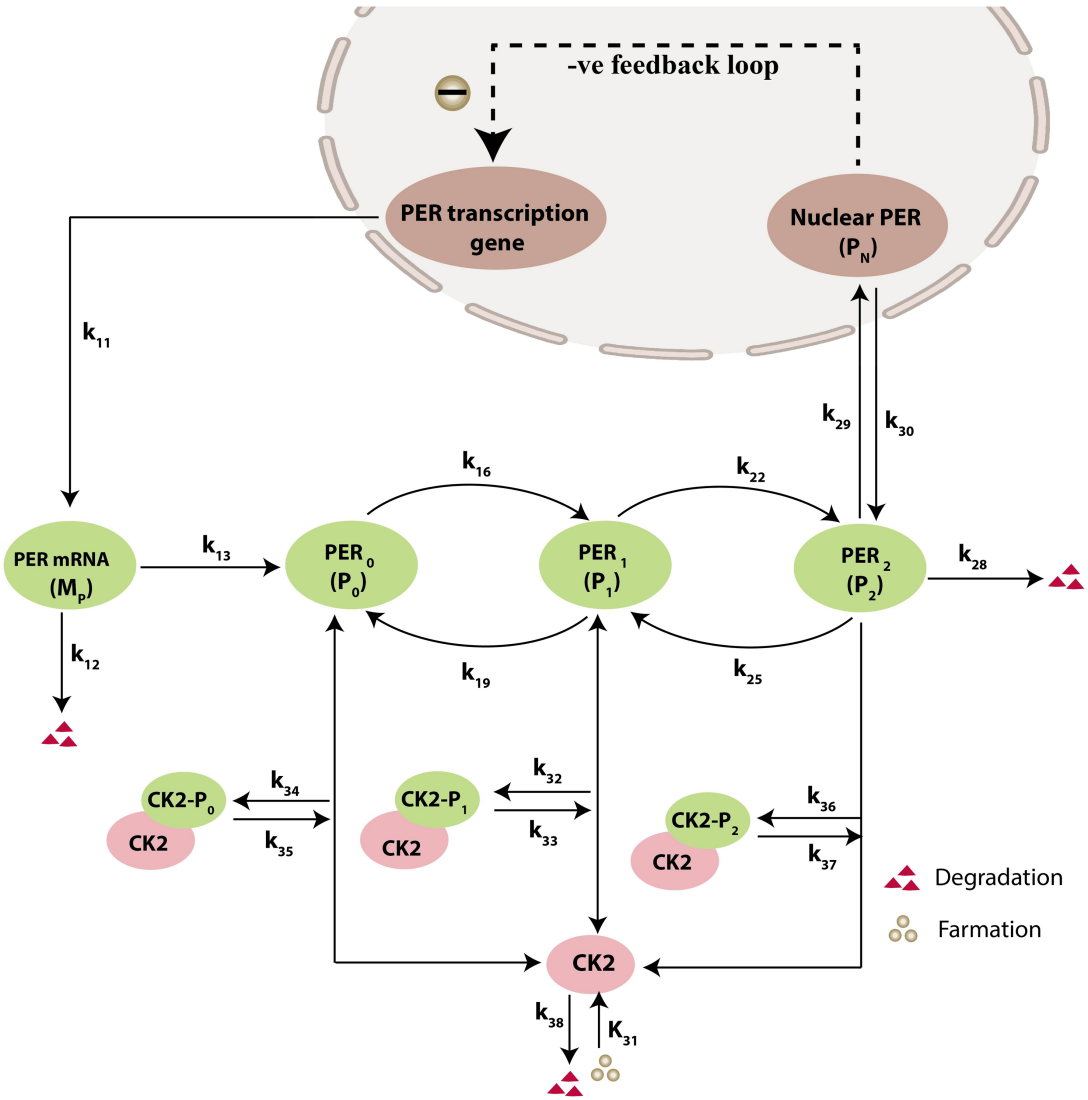
Schematic diagram of circadian rhythm model driven by CK2. The model depicts a phenotype for molecular mechanism of circadian oscillations based on negative autoregulation of gene expression. The red triangle represents the degradation and green circle represents the formation.

Prompted by published experimental reports, which indicate that CK2 protein interacts with clock proteins in circadian rhythm (Yu et al., 2006; Szabó et al., 2013), we incorporated the molecular signaling pathways of CK2 in our model. The proposed schematic circadian–CK2 integrative model (Figure 1) is an extension of the circadian rhythm model in Drosophila, reported by Gonze et al. (2002) and Albrecht (2010). This model is based on the inhibition of a nuclear clock protein ( $P_{N}$ ) at the level of transcription of its gene into mRNA ( $M_{P}$ ) (Albrecht, 2010). mRNA is synthesized in the nucleus and is transferred to the cytosol, where it accumulates at a maximum rate of $k_{9}$ and gets ubiquitinated by enzymes (E1, E2, E3, and E4) with a rate of $k_{12}$ . In our model, the spatial temporal fluctuations in the concentrations of various forms of the nuclear clock protein ( $P_{N}$ ) or cytosolic regulatory protein ( $P_{0},P_{1}$ and $P_{2}$ ) is governed by the biochemical pathways. The rate of synthesis of protein $P_{0}$ is proportional to the formation of $M_{P},$ and is characterized by an apparent first order rate constant $k_{13}$ . Parameters $k_{16}\;$ and $k_{22}$ denote the maximum rate(s) and Michaelis constant(s) of the phosphatase and kinase involved in the reversible phosphorylation of $P_{1}$ into $P_{2}$ and $P_{2}$ into $P_{1}$ , respectively. The fully phosphorylated state $\left(P_{2}\right)$ is degraded by CK2 and is transported into the nucleus at a rate characterized by the apparent first-order rate constant $k_{20}.$ Transport of the $P_{N}$ into the cytosol is characterized by the apparent first-order rate constant $k_{30}$ . It is reported that the negative feedback is exerted by the nuclear clock protein on gene transcription. CK2 phosphorylates the clock protein, which may form three complexes, namely $\mathrm{CK}2-P_{0}$ , $\mathrm{CK}2-P_{1},$ and $\mathrm{CK}2-P_{2,}$ due to three different forms of the available proteins. Synthesis of $\mathrm{CK}2-P_{0}$ is assumed to occur with a rate constant of $k_{32,}$ and subsequently, the dissociation of this complex is assumed to occur with a rate constant of $k_{33}$ . In a similar manner, it is hypothesized that complex formation of $\mathrm{CK}2-P_{1}$ occurs with a rate constant of $k_{34}$ and its dissociation follows with a rate constant of $k_{35}$ . Further, complex formation of CK2 − P2 is considered to occur with a rate constant of $k_{36},$ and subsequently, the dissociation of this complex is assumed to occur with rate constants of $k_{37}$ . CK2 is ubiquitinated at a rate constant of $k_{38}$ . Synthesis of CK2 in the network is assumed to occur at the rate constant of $k_{38}$ . CK2 plays a significant role in various processes of the organism such as biological clocks. Table 1 provides the list of clock proteins linked to the integrated model and Table 2 presents the list of the biochemical reactions and propensity functions (probability of reaction). The rate constants associated with the proposed model are presented in Table 3.

**Table 1:** List of molecular species used in our study.

S. No.	Molecular species	Description
1	$P_{N}$	Nuclear PER protein
2	G	Promoter of the gene without ligand
3	$G_{P_{N}}$	Complex of G and one molecule of $P_{N}$
4	$G_{P_{2}}$	Complex of G and two molecules of $P_{N}$
5	$G_{P_{3}}$	Complex of G and three molecules of $P_{N}$
6	$G_{P_{4}}$	Complex of G and four molecules of $P_{N}$
7	$M_{P}$	ER mRNA
8	$E_{m}$	The enzyme degradate PER mRNA
9	$C_{m}$	Complex of PER mRNA and enzyme EM
10	$P_{0}$	Unphosphorylated PER protein
11	$P_{1}$	De-phosphorylatyed PER protein
12	$P_{2}$	Phosphorylatyed PER protein
13	$E_{1}$	The enzyme phosphorylates $P_{0}$ protein into $P_{1}$
14	$C_{1}$	Complex of PER ( $P_{0}$ ) protein and enzyme $E_{1}$
15	$E_{2}$	The enzyme de-phosphorylate $P_{1}$ protein into $P_{0}$
16	$C_{2}$	Complex of $P_{1}$ protein and enzyme $E_{2}$
17	$E_{3}$	The enzyme phosphorylates $P_{1}$ protein into $P_{2}$
18	$C_{3}$	Complex of $P_{1}$ protein and enzyme $E_{3}$
19	$E_{4}$	The enzyme de-phosphorylate $P_{2}$ protein into $P_{1}$
20	$C_{4}$	Complex of $P_{2}$ protein and enzyme $E_{4}$
21	$E_{d}$	Th Enzyme degradation the phosphorylated $P_{2}$
22	$C_{d}$	Complex of $P_{2}$ protein and enzyme $E_{1}$
23	CK2	Casein kinase 2
24	$CK2-P_{0}$	Complex of CK2 and $P_{0}$
25	$CK2-P_{1}$	Complex of CK2 and $P_{1}$
26	$CK-P_{2}$	Complex of CK2 and $P_{2}$

**Table 2:** The mathematical model for circadian rhythms.

S. No.	Chemical reaction	Probability of reaction
1	$G+P_{N}\overset{E_{1}}{\to }\;GP_{N}$	*u*_1_ = *k*_1_ × *G* × *P_N_ /V*
2	$GP_{N}\;\overset{E_{2}}{\to }\;G+P_{N}$	*u*_2_ = *k*_2_ × *GP_N_*
3	$G+PN\overset{E_{3}}{\to }\;GPN2$	*u*_3_ = (*k*_3_ × *GP_N_* × *P_N_*)/*V*
4	$GPN2\;\overset{E_{4}}{\to }\;GPN+PN$	*u*_4_ = *k*_4_ × *GP*_N2_
5	$GPN2+PN\overset{k_{5}}{\to }\;GPN3$	*u*_5_ = (*k*_5_ × *GP*_N2_ × *P_N_*)*/V*
6	$GPN3\;\overset{E_{6}}{\to }GPN2+PN3$	*u*_6_ = *k*_6_ × *GP*_N3_
7	$PN3+PN\;\overset{E_{7}}{\to }\;GPN4$	*u*_7_ = (*k*_7_ × *GP_N_* 3 × *P_N_*)*/V*
8	$GPN4\;\overset{E_{8}}{\to }\;GPN3+PN$	*u*_8_ = *k*_8_ × *GP*_N4_
9	$\left[G,GPN,GPN2,GPN3\right]\overset{E_{9}}{\to }\;MP$	*u*_9_ = *k*_9_ × (*G, GP_N_, GP*_N2_*, GP*_N3_)
10	$MP+Em\;\overset{E_{10}}{\to }\;Cm$	*u*_10_ = (*k*_10_ × *M_P_* × *E_m_*)*/V*
11	$Cm\;\overset{E_{11}}{\to }\;MP+Em$	*u*11 = *k*11 × *Cm*
12	$Cm\;\overset{E_{12}}{\to }\;Em$	*u*12 = *k*12 × *Cm*
13	$MP\overset{E_{13}}{\to }\;MP+P0$	*u*13 = *k*13 × *MP*
14	$P0+E1\;\overset{E_{14}}{\to }\;C1$	*u*_14_ = (*k*_14_ × *P*_0_ × *E*_1_)*/V*
15	$C1\overset{15}{\to }EP0+E1$	*u*15 = *k*15 × *C*1
16	$C1\;\overset{E_{16}}{\to }\;P1+E1$	*u*16 = *k*16 × *C*1
17	$P1+E2\;\overset{E_{17}}{\to }\;C2$	*u*_17_ = *k*_17_ × *P*_1_ × *E*_2_*/V*
18	$C2\;\overset{E_{18}}{\to }\;P1+E2$	*u*18 = *k*18 × *C*2
19	$C2\;\overset{E_{19}}{\to }\;P0+E2$	*u*19 = *k*19 × *C*2
20	$P1+E3\;\overset{E_{20}}{\to }\;C3$	*u*_20_ = (*k*_20_ × *P*_1_ × *E*_3_)*/V*
21	$C3\;\overset{E_{21}}{\to }\;P1+E3$	*u*21 = *k*21 × *C*3
22	$C3\;\overset{E_{22}}{\to }\;P2+E3$	*u*22 = *k*22 × *C*3
23	$P2+E4\;\overset{E_{23}}{\to }\;C4$	*u*_23_ = *k*_23_ × *P*_1_ × *E*_4_*/V*
24	$C4\overset{E_{2}}{\to }\;P2+E4$	*u*24 = *k*24 × *C*4
25	$C4\;\overset{E_{25}}{\to }\;P1+E4$	*u*25 = *k*25 × *C*4
26	$P2+Ed\;\overset{E_{26}}{\to }\;Cd$	*u*_26_ = (*k*_26_ × *P*_2_ × *E_d_*)*/V*
27	$Cd\;\overset{E_{27}}{\to }\;P2+Ed$	*u*27 = *k*27 × *Cd*
28	$Cd\;\overset{E_{28}}{\to }\;Ed$	*u*28 = *k*28 × *Cd*
29	$P2\;\overset{E_{29}}{\to }\;PN$	*u*29 = *k*29 × *P*2
30	$PN\;\overset{E_{30}}{\to }\;P2$	*u*30 = *k*30 × *PN*
31	$\varphi \;\overset{E_{31}}{\to }\;CK2$	*u*31 = *k*31
32	$CK2+P0\;\overset{E_{32}}{\to }\;CK2-P0$	*u*_32_ = *k*_34_ × *CK*2 × *P*_0_
33	$CK2-P0\;\overset{E_{33}}{\to }\;CK2+P0$	*u*_33_ = *k*_33_ × *CK*2 − *P*_0_
34	$CK2+P1\;\overset{E_{34}}{\to }\;CK2-P1$	*u*_34_ = *k*_34_ × *CK*2 × *P*_1_
35	$CK2-P1\;\overset{E_{35}}{\to }\;CK2+P1$	*u*_35_ = *k*_35_ × *CK*2 − *P*_1_
36	$CK2+P2\;\overset{E_{36}}{\to }\;CK2-P2$	*u*_36_ = *k*_36_ × *CK*2 × *P*_2_
37	$CK2-P2\;\overset{E_{37}}{\to }\;CK2+P2$	*u*_37_ = *k*_37_ × *CK*2 − *P*_2_
38	$CK2\;\overset{E_{38}}{\to }\;\varphi$	*u*_38_ = *k*_38_ × *CK*2

The first column lists the chemical reaction channels. The second column lists the propensity function (probability of reaction) of occurrence of the reaction steps; kinetic constants related to bimolecular reactions are scaled by V . In the developed stochastic model, when varying V to modify the number of molecules involved in the circadian oscillatory mechanism, we tend to keep at least one molecular species without altering the relative weights of the different probabilities $u_{i}$ .

**Table 3:** Parameters used in numerical simulations of the stochastic model for circadian rhythms.

S. No.	Parameter	Description	References
1	*k*_1_ = *V mol*^−1^ *h*^−1^	Rate of association constant of G and *P_N_*	Gonze et al. (2002) and Goldbeter (2002)
2	*k*_2_ = (160 × *V*)*h*^−1^	Rate of dissociation constant of G and *P_N_*	Gonze et al. (2002) and Goldbeter (2002)
3	*k*_3_ = (10 × *V*)*mol*^−1^ *h*^−1^	Rate of association constant of G and *P_N2_*	Gonze et al. (2002) and Goldbeter (2002)
4	*k*_4_ = (100 × *V*)*h*^−1^	Rate of dissociation constant of G and *P_N2_*	Gonze et al. (2002) and Goldbeter (2002)
5	*k*_5_ = 100 *V mol*^−1^ *h*^−1^	Rate of association constant of G and *P_N3_*	Gonze et al. (2002) and Goldbeter (2002)
6	*k*_6_ = (10 × *V*)*h*^−1^	Rate of dissociation constant of G and *P_N3_*	Gonze et al. (2002) and Goldbeter (2002)
7	*k*_7_ = 100 *V mol*^−1^ *h*^−1^	Rate of association constant of G and *P_N4_*	Gonze et al. (2002) and Goldbeter (2002)
8	*d*_8_ = (10 × *V*)*h*^−1^	Rate of dissociation constant of G and *P_N4_*	Gonze et al. (2002) and Goldbeter (2002)
9	*k*_9_ = (0.5 × *V*)*mol*^−1^ *h*^−1^	Translation rate of *M_P_*	Gonze et al. (2002) and Goldbeter (2002)
10	*k*_10_ = 165 *mol*^−1^ *h*^−1^	Rate of binding constant of *M_P_* to enzyme *E_m_* to form complex *C_m_*	Gonze et al. (2002) and Goldbeter (2002)
11	*k*_11_ = 30 *h*^−1^	Rate of dissociation constant of complex of *C_m_* to *M_P_* and enzyme *E_m_*	Gonze et al. (2002) and Goldbeter (2002)
12	*k*_12_ = 3 *h*^−1^	Rate of catalytic decomposition of *C_m_*	Gonze et al. (2002) and Goldbeter (2002)
13	*k*_13_ = 2 *h*^−1^	Rate of synthesis of the *P*_0_, proportional to *M_P_*	Gonze et al. (2002) and Goldbeter (2002)
14	*k*_14_ = 146.6 *mol*^−1^ *h*^−1^	Rate of binding constant of unphosphorylated *P*_0_ to enzyme *E*_1_ to form complex *C*_1_	Gonze et al. (2002) and Goldbeter (2002)
15	*k*_15_ = 200 *h*^−1^	Rate of dissociation constant of complex of *C*_1_ to *P*_0_ and enzyme *E*_1_	Gonze et al. (2002) and Goldbeter (2002)
16	*k*_16_ = 20 *h*^−1^	Dissociation constant of complex *C*_1_ into Phosphorylated *p*_1_ and *E*_1_ Phosphorylation of *p*_1_	Gonze et al. (2002) and Goldbeter (2002)
17	*k*_17_ = 82.5 *mol*^−1^ *h*^−1^	Rate of binding constant of phosphorylated *P*_1_ and enzyme *E*_2_ to form complex *C*_2_	Gonze et al. (2002) and Goldbeter (2002)
18	*k*_18_ = 150 *h*^−1^	Rate of dissociation constant of complex of *C*_2_ to *P*_1_ and enzyme *E*_2_ (De-phosphorylation of *P*_1_)	Gonze et al. (2002) and Goldbeter (2002)
19	*k*_19_ = 15 *h*^−1^	Rate of dissociation constant of complex of *C*_2_ to *P*_0_ and enzyme *E*_2_ (Dephosphorylation of *P*_0_)	Gonze et al. (2002) and Goldbeter (2002)
20	*k*_20_ = 146.6 *mol*^−1^ *h*^−1^	Rate of binding constant of phosphorylated *P*_1_ to enzyme *E*_3_ to form complex *C*_3_	Gonze et al. (2002) and Goldbeter (2002)
21	*k*_21_ = 200 *h*^−1^	Dissociation constant of complex of *C*_3_ to *P* an enzyme *E*_3_ (De-phosphorylation/ubiquitous of *P*_1_)	Gonze et al. (2002) and Goldbeter (2002)
22	*k*_22_ = 20 *h*^−1^	Dissociation constant of complex of *C*_3_ to *P*_2_ and enzyme *E*_3_ (De-phosphorylation/ ubiquitous of *P*_2_)	Gonze et al. (2002) and Goldbeter (2002)
23	*k*_23_ = 82.5 *mol*^−1^ *h*^−1^	Rate of binding constant of phosphorylated *P*_2_ to enzyme *E*_4_ to form complex *C*_4_	Gonze et al. (2002) and Goldbeter (2002)
24	*k*_24_ = 150 *h*^−1^	Dissociation constant of complex of *C*_4_ to *P*_2_ and enzyme *E*_3_ (De- phosphorylation/ubiquitous of *P*_2_)	Gonze et al. (2002) and Goldbeter (2002)
25	*k*_25_ = 15 *h*^−1^	Rate of dissociation constant of com- plex of *C*_4_ to *P*_1_ and enzyme *E*_4_ (De-phosphorylation/ubiquitous of *P*_1_)	Gonze et al. (2002) and Goldbeter (2002)
26	*k*_26_ = 1,650 *mol*^−1^ *h*^−1^	Rate of binding constant of phosphorylated *P*_2_ to enzyme *E_d_* to form complex *C_d_*	Gonze et al. (2002) and Goldbeter (2002)
27	*k*_27_ = 150 *h*^−1^	Rate of dissociation constant of com- plex of *C_d_* to *P*_2_ and enzyme *E_d_* (De-hosphorylation/ubiquitous of *P_d_*)	Gonze et al. (2002) and Goldbeter (2002)
28	*k*_28_ = 15 *h*^−1^	Rate of catalytic decomposition of *C_d_*	Gonze et al. (2002) and Goldbeter (2002)
29	*k*_29_ = 2 *h*^−1^	Rate of transportation of *P*_2_ into *P_N_* from cy- tosol to nucleus	Gonze et al. (2002) and Goldbeter (2002)
30	*k*_30_ = 1 *h*^−1^	Rate of transport *P_N_* from nucleus to cytosol (negative feedback of cooperative nature on the expression of *P_N_* gene)	Gonze et al. (2002) and Goldbeter (2002)
31	*k*_31_ = 0.001–1.0 *mol*^−1^ *h*^−1^	Rate of CK2 synthesis	Estimated
32	*k*_32_ = 100 *mol*^−1^ *h*^−1^	Rate of complex formation of CK2 and phos- phorylated *P*_0_	Szabó et al. (2013)
33	*k*_33_ = 15 *mol*^−1^ *h*^−1^	Rate of dissociation of CK2 and *P*_0_	Szabó et al. (2013)
34	*k*_34_ = 100 *mol*^−1^ *h*^−1^	Rate of complex formation of CK2 and phos- phorylated *P*_1_	Szabó et al. (2013)
35	*k*_35_ = 15 *mol*^−1^ *h*^−1^	Rate of dissociation of CK2 and *P*_1_	Szabó et al. (2013)
36	*k*_36_ = 100 *mol*^−1^ *h*^−1^	Rate of complex formation of CK2 and phos- phorylated *P*_2_	Szabó et al. (2013)
37	*k*_37_ = 15 *mol*^−1^ *h*^−1^	Rate of dissociation of CK2 and *P*_2_	Szabó et al. (2013)
38	*k*_38_ = 0.5 *mol*^−1^ *h*^−1^	Rate of degradation of CK2	Prediction by theoretical experimental

### Technique for simulation of the biochemical reaction pathway

Complex dynamical processes, governed by a set of well-defined reaction channels, are generally noise-induced stochastic processes due to random molecular interactions in the system (origin of intrinsic noise) and continuous interaction of the system with random environmental fluctuations (origin of extrinsic noise) (Doob, 1942; Gillespie, 1977; Miyazaki et al., 2004). The system with N clock protein variables, whose population vector is defined by, $\vec{X}\;\left(t\right)={\left(X_{1},X_{2},.\;\;..\;\;,X_{N}\right)}^{T}$ , where T is the transpose of the vector, which undergo M reaction channels, $R_{I},i=1,2,.\;\;..\;\;,M$ is given by,


(1) $$R_{i}:a_{1}\;X_{1}+a_{2}\;X_{2}+\cdots +a_{N}\;X_{N}\;\overset{k_{i}}{\to }b_{1}\;X_{1}+b_{2}\;X_{2}+\cdots +b_{N}\;X_{N}$$

where $\left\{k_{i}\right\},i=1,2,.\;\;..\;\;,M$ is the set of classical rate constants. The stochastic rate constant $c_{i}\;$ of any of its reactions can be expressed in terms of classical rate constant $k_{i}$ as $c_{i}=k_{i}V^{1-\nu }$ , where V is the system size and ν is the state change parameter of its reaction (Gillespie, 1977; Gillespie, 2000). The trajectories of the variables provided by birth and death processes due to molecular interaction given by the Equation 1 can be traced by solving the master equation, for the rate of change of configurational probability $P\left(\vec{X},t\right)$ as a function of time. The master equation is denoted as


(2) $$\frac{\partial P\left(\vec{X},t\right)}{\partial t}=\underset{\left\{\vec{X}\right\}}{\sum }W_{\vec{X}\prime \to \vec{X}}\;P\left(\vec{X},t\right)+\underset{\left\{\vec{X}\prime \right\}}{\sum }W_{\vec{X}\prime \to \vec{X}}\;P\left({\vec{X}}^{\prime },t\right)$$

where W and $W^{\prime }$ are the transition probabilities of the two configurational states $\vec{X}$ and { $\vec{X\;}$ ′}. Solving the master Equation 2 analytically for complex biological process is extremely difficult. However, numerical solutions for the master Equation 2 can be derived using stochastic simulation algorithm (SSA) (Gillespie, 1977; Gillespie, 2000) that is based on the theoretical foundations developed by Doob (1942) and initially proposed by Kendall (1950). The stochastic simulation implements a Monte Carlo algorithm that provides the exact numerical solution by considering every possible interaction in the system (Gillespie, 1977; Singh et al., 2018). This algorithm is in deed a non-spatial, individual-based analog of the master Equation 2, which is constructed on the physical basis of molecular collision in each reaction channel at a certain constant temperature.

This SSA is based on two crucial independent random processes, namely reaction fire and reaction time. These two processes are implemented in this algorithm by generating two statistically independent random numbers, namely $r_{1}$ and $r_{2,}$ such that the reaction time is computed using $\tau =-\frac{1}{a_{0}}\ln r_{1}\;$ , where $a_{0}\underset{i}{\sum }a_{i}$ , $a_{i}$ is the $i^{th}$ propensity function given by $a_{i}=h_{i}c_{i}$ , where $h_{i}$ is the number of possible molecular combinations of $i^{th}$ reaction, and the $k^{th}\;$ reaction will fire when it satisfies $\overset{k}{\underset{i=1}{\sum }}a_{i}\leq a_{0}r_{2}<\overset{k+1}{\underset{i=1}{\sum }}a_{i}$ . Intrinsic noise ( ξ ) associated with the clock protein dynamics in the system is inversely proportional to the square root of the systems size V (i.e., $\xi \propto \frac{1}{\sqrt{V}}\;)$ (Gillespie, 2000; Singh et al., 2018; Sharma et al., 2019; Singh et al., 2021).

### Algorithm to calculate permutation entropy: the Bandt and Pompe approach

Permutation entropy can be used to measure the complexity of a system associated with the dynamics of the system’s variables (Bandt and Pompe, 2002). The basic algorithm for calculating permutation entropy (H ) of a time series is as follows:

Consider a dynamical variable x(t) of a system given by the discrete time series $=\left\{x_{i}\right\},$ i=1,2,...,N , where N is the finite total number of discrete time elements in the time series data. We define an embedding dimension (d ), preferably d=3,...,7, to represent the data in consecutive patterns of the size dimension d . For a particular value of d , there are M possible permuted sequences of inequalities of sequence elements. If we take d=3 , we will have M=6 arrangements of permutations given by,

$u_{1}=\left\{x_{1},x_{2},x_{3}\right\}$ , $u_{2}=\left\{x_{1},x_{3},x_{2}\right\}$ , $u_{3}=\left\{x_{2},x_{1},x_{3}\right\}$ , $u_{4}=\left\{x_{2},x_{3},x_{1}\right\}$ , $u_{5}=\left\{x_{3},x_{1},x_{2}\right\}$ , $u_{6}=\left\{x_{3},x_{2},x_{1}\right\}$ , where, $x_{1}\neq x_{2}\neq x_{3}.$ Then, we calculate the probability $\left(p_{u}\right)$ for each single permutation sequence, which is the ratio of number of values for a particular sequence of permutations to the total number of all possible permutations for the embedding dimension, d, in the time series data. Shannon entropy for a sequence of perturbations can then be calculated by Equation 3,


(3) $$H_{u}=-\overset{d}{\underset{i=1}{\sum }}p_{i}\log\left(p_{i}\right)$$

and permutation entropy of embedded dimension (d) by the sum of these entropies as mentioned in Equation 4,


(4) $$H=\frac{1}{\log\left(M\right)}\overset{M}{\underset{u=1}{\sum }}H_{u}$$

where 0≤H≤1 . The mapped permutation entropy spectrum of time series x(t) is indicated by $H=\left\{H_{1},H_{2},.\;\;..\;\;,H_{N}\right\}$ , and exhibits a behavior similar to that of Lyapunov spectrum of the same time series (Bodine et al., 2001).

## Results

We performed large-scale numerical simulations of the proposed circadian–CK2 integrated model developed using stochastic simulation algorithm (SSA) (Gillespie, 1977). We demonstrate the SSA with a primary focus on the regulatory role of CK2 in the dynamics of clock proteins involved in circadian rhythm. We also demonstrate the impact of molecular noise owing to molecular integration of environment within the system (Goldbeter et al., 2017).

### Modulation of clock proteins ( $P_{N}$ , $M_{P}$ ) by CK2

**Figure 2:**
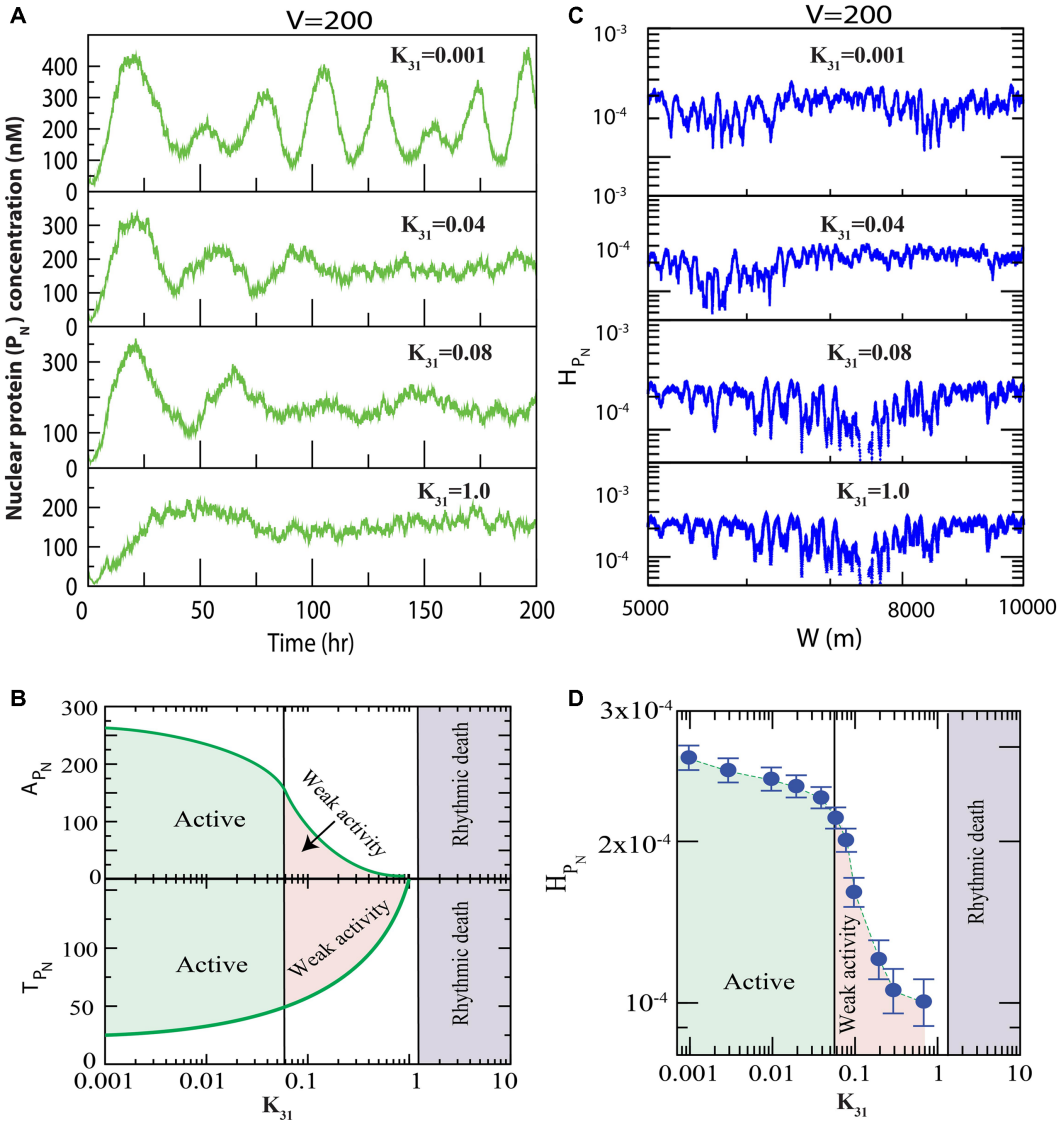
Dynamics of variables of nuclear PER proteins (P_N_) in circadian rhythm driven by casein kinase 2 (CK2). We consider all possible interactions of CK2 with PER_0_ (P_0_), PER_1_ (P_1_) and PER_2_ (P_2_) as shown in Figure 1. **(A)** Plots of dynamics of $P_{N}$ for four values of synthesis rate of CK2 ( $k_{31}$ ) for fixed value of system’s size *V* = 200. **(B)** Plots of amplitude of oscillation ( $A_{P_{N}})$ and time period of oscillation ( $T_{P_{N}})$ as a function of synthesis rate, k_31,_ of CK2 kinase. Each point in the curves represents average of the amplitude and time period in each time series between [10–200] hours corresponding to values of $k_{31}$ . Based on the behavior of the system, three different circadian states, namely, active, weak activity and rhythmic death, are identified. Variations in expressions of CK2 might cause changes in the interaction rate of P_N_ in our proposed model. We observed that the variations in the dynamics of the P_N_ clock proteins are driven by CK2 via changes in the values for $k_{31}$ . If CK2 expression is small ( $0.001\geq k_{31}<0.01$ ), the system displays prominent oscillation (active state) and the amplitude of oscillation is $A_{P_{N}}∼285\pm 3.$ Further increases in $k_{31}$ ( $k_{31}=0.04,0.08)\;$ suppressed the oscillation (weak activity ), and decreased amplitude ( $A_{P_{N}}∼150\pm 5$ ). When the $k_{31}$ is sufficiently large ( $k_{31}\sim 1.0$ ), the $P_{N}$ dynamics displayed rhythmic death for both oscillation and amplitude. **(C)** Permutation entropy spectrum of $H_{P_{N}}$ as a function of W (m) for the corresponding time series on $P_{N}$ . **(D)** Permutation entropy curves as a function of $k_{31}$ with error bars with the three circadian states clearly demarcated. If CK2 expression is small ( $0.001\geq k_{31}<0.01$ ), the system displays prominent oscillation (active state) and large permutation entropies ( $H_{P_{N},}^{A}>0.0003$ ). Further increases in $k_{31}$ ( $k_{31}=0.04,0.08)\;$ suppressed the oscillation (weak activity ) and decreased permutation entropies ( $0.0003>H_{P_{N},}^{WA}>0.0001$ ). When $k_{31}$ is sufficiently large ( $k_{31}\sim 1.0$ ), the $P_{N}$ dynamics showed less values for both the oscillation and permutation entropies ( $H_{P_{N},}^{RD}<0.0001$ ).

We first present the results of how CK2 regulates the dynamics of clock protein ( $P_{N}$ , $M_{P}$ ). Since the population of the CK2 protein in the system of size V (at fixed V=200) is proportional to the rate of synthesis of this protein in the system ( $\mathrm{CK}2\propto k_{31}$ ), variation in CK2 might cause changes in the interaction rate of other clock proteins in the reaction network model. Hence, we looked for variations in the dynamics of the clock proteins, driven by CK2 *via* changes in the values for $k_{31}$ . First, we allowed all three phosphorylation events in the cytosolic PER proteins (well-known clock proteins), $P_{0},P_{1},P_{2},$ with CK2 to occur (Figure 1), and found that for small values of $k_{31}=0.001$ , prominent oscillation (active state) in the nuclear PER protein is exhibited with time period of oscillation, $T_{P_{N}}\sim 23.4\pm 0.13$ h and amplitude of oscillation $A_{P_{N}}∼285\pm 3\;$ (Figure 2A upper panel). Further increase in $k_{31}$ suppressed the oscillation ( $k_{31}=0.04,0.08),$ allowing increased $T_{P_{N}}$ and decreased $A_{P_{N}}$ significantly, which could correspond to weak circadian activity (Crino, 2011). This increase in the time period was due to increased phosphorylation owing to an increase in interaction of CK2 with the PER protein ( $P_{0},P_{1},P_{2}$ ), which was evident from the experimental reports of Cao et al. (2009). Further, weak circadian activity may cause various diseases, such as aging of the brain, metabolic dysfunction, dementia, and cancer-related disorders (Rao et al., 2002). When $k_{31}$ is sufficiently large ( $k_{31}\sim 1.0$ ), the $P_{N}$ dynamics showed both oscillation and amplitude death scenarios (Rao et al., 2002). This state may correspond to circadian rhythm death (Rao et al., 2002). These three important states in the dynamics of $P_{N}$ of the circadian rhythm induced by CK2 can be shown in two-dimensional space of parameter as ( $k_{31},A_{P_{N}}$ and $k_{31},T_{P_{N}}$ ) (Figure 2B). Here, the parameters $A_{P_{N}}$ and $T_{P_{N}}$ are mean values of $P_{N}$ amplitude and time period for a range of t=[10−500] hours. In the phase diagram presented in Figure 2B, all three circadian states are seen to be clearly demarcated.

We delineated the measure of complexity in the three derived states (active, weak activity, and rhythmic death) driven by CK2 by calculating permutation entropy ( $H_{P_{N}})$ of the dynamics of $P_{N}$ at the three respective states (Figure 2C). Upon considering the average permutation entropies of active, weak activity, and rhythmic death as $H_{P_{N},}^{A}\;H_{P_{N}}^{WA},$ and $H_{P_{N}}^{RD},$ the simulation results demonstrated that $H_{P_{N}}^{A}>H_{P_{N}}^{WA}>H_{P_{N}}^{RD}$ . The three circadian rhythm states (active, weak activity, and rhythmic death) could easily be classified using $H_{P_{N}}^{A}$ (Figure 2D). In this phase diagram, each point is the average of permutation spectrum for each value of $k_{31}$ with error bars. Hence, the results indicated that $H_{P_{N}}$ could be used as a parameter to classify various states of the circadian rhythm.

**Figure 3:**
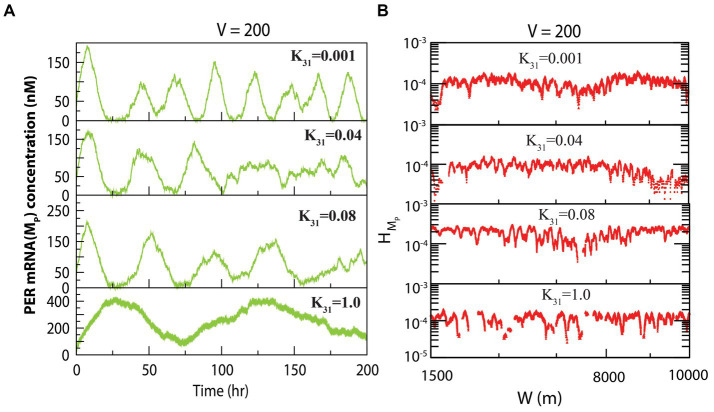
Dynamics of variables of PER mRNA ( $M_{P}$ ) in circadian rhythm model driven by CK2. **(A)** Plots of dynamics of $M_{P}$ for four values of synthesis rate of CK2 ( $k_{31}$ ) for a fixed value of system’s size *V* = 200. **(B)** Permutation entropy spectrum plots of $H_{M_{P}}$ as a function of W(m) for the corresponding time series on $M_{P}$ .

The dynamics of $M_{P}$ exhibited similarity to that of $P_{N}$ at fixed system size and variable concentration of CK2. The dynamics of $M_{P}$ for various values of $k_{31}$ for constant values of system size is presented in Figure 3A. We measured the permutation entropies of *MP* at the three states under the situation of fixed system size and of variations of the CK2 concentration. In the case of permutation entropies of *MP* at the three states (Figure 3B), we made observations similar to that of $P_{N}$ (as depicted in Figure 2B).

We propose that $H_{P_{N}}$ and $H_{M_{P}}$ can be useful for clinicians and medical practitioners to classify the various states of circadian rhythm. These results suggest that CK2 plays a very dynamic role in the regulation of circadian rhythm in living systems. The change in rhythmic properties may alter the physiological processes of the organism leading to various diseases.

### Impact of CK2 configurational interactions with the per proteins on circadian rhythm

**Figure 4:**
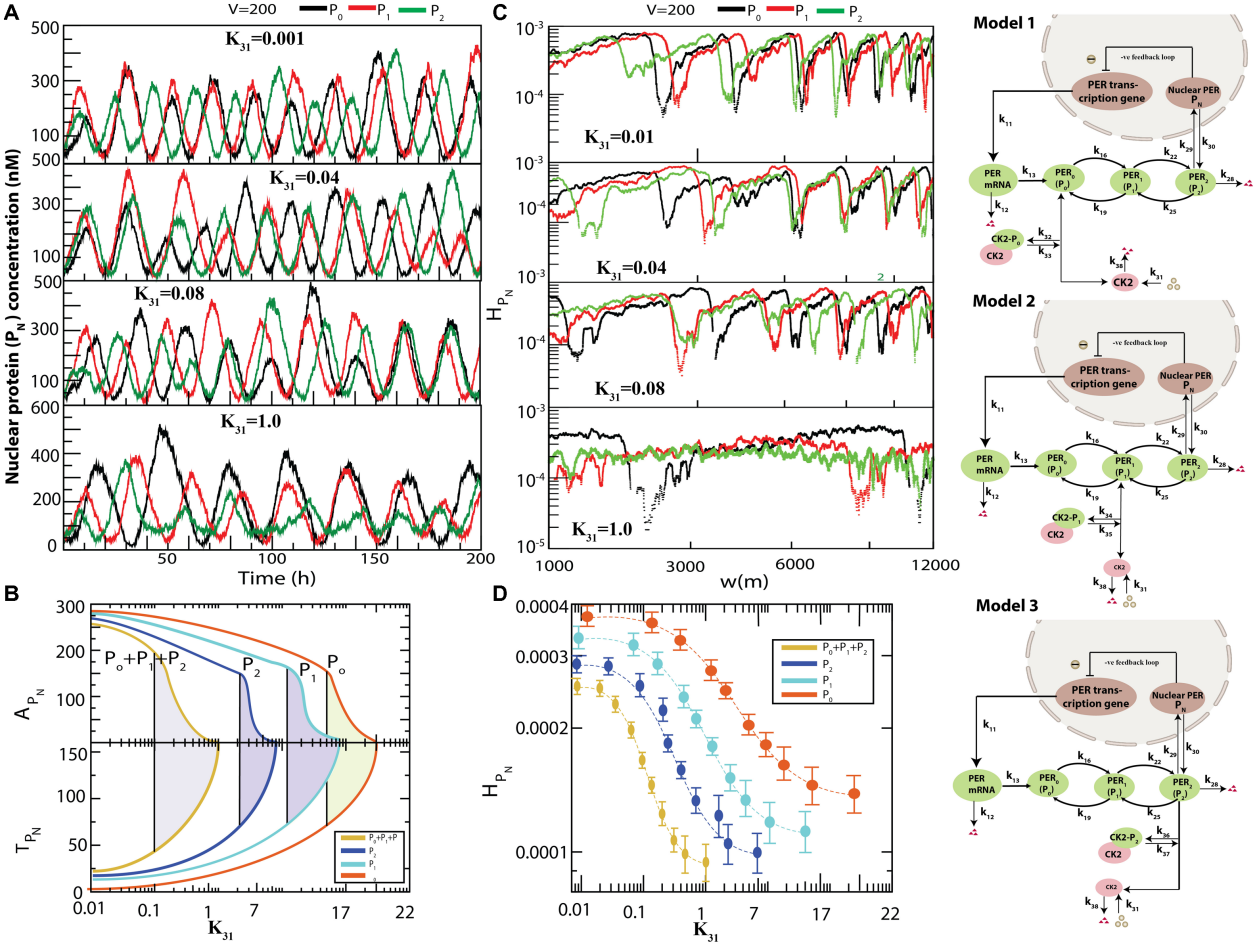
Different configurational interaction of CK2 with *PER* gene mutants. **(A)** Dynamics of nuclear PER protein ( $P_{N}$ ) for different values of CK2 synthesis rate ( $k_{31}$ ) when CK2 interacts with different PER protein ( $P_{0}$ , $P_{1}$ , and $P_{2}$ ). **(B)** The phase diagram like behavior of circadian rhythm driven by CK2 – Plots of $A_{P_{N}}$ and $T_{P_{N}}$ as a function of CK2 for all four configurational interactions of CK2 with $P_{0}$ , $P_{1}$ , and $P_{2}$ . The model for interaction of $P_{0}$ , $P_{1}$ and $P_{2}$ are given in model 1, model 2 and model 3, respectively. **(C)** Corresponding permutation entropy spectrum plot of $H_{P_{N}}$ as a function of *W* (*m*) for the corresponding time series on $P_{N}$ . **(D)** Permutation entropy $H_{P_{N}}$ with respect to $k_{31}$ for the four possible configurational interactions of CK2 with $P_{0}$ , $P_{1},$ $P_{2}\;and\;P_{0}$ $+P_{1}+P_{2}$ .

Figure 4 presents the various possible interactions of CK2 with each of the PER proteins $(P_{0}$ , $P_{1},$ and $P_{2})$ to form complexes $\mathrm{CK}2-P_{0}$ , $\mathrm{CK}2-P_{1}$ , and $\mathrm{CK}2-P_{2}$ . All simulations were performed for the same values of $k_{31}\;$ and V . used in the previous simulation depicted in Figure 3. The results demonstrated that if CK2 interacts individually with any one of the PER proteins *via* formation of a complex, $P_{2}\;$ dynamics exhibits both active (for smaller values of $k_{31}$ ) and weak activity (for larger values of $k_{31}$ ) states (Figure 4A). However, the system needs significantly large values of $k_{31}$ to obtain the rhythmic death state (data not shown). Further, configurational interaction of CK2 with $P_{2}$ is much more sensitive in driving the circadian rhythm states, as compared to those with $P_{1}$ or $P_{0}$ . We then calculated $A_{P_{N}}$ and $T_{P_{N}}$ for each time series when CK2 was allowed to interact with each one of $P_{0}$ , $P_{1},$ and $P_{2}$ in the circadian–CK2 model (Figure 4B). Each curve in $A_{P_{N}}$ and $T_{P_{N}}$ (Figure 4B) illustrates that the three circadian rhythm states could be observed. We also found that interaction of CK2 with $P_{2}\;$ is more sensitive than that with either $P_{0}$ or $P_{1}$ because the three circadian states are found at smaller values of $k_{31}\;$ in the case of $\mathrm{CK}2-P_{2}\;\mathrm{complex}$ .

The three models, namely model1, model2, and model3, as presented in Figure 4, are patterns of weak activity when CK2 systematically interacts with any one of the PER proteins ( $P_{0},P_{1,}\;\mathrm{and}\;P_{2}$ ); these three patterns can also be considered as pathological states of the corresponding configuration. This rationale is acceptable because the patterns of CK2 activities (namely amplitude and time period) are significantly changed due to drastic increase in phosphorylation of PER protein(s) with CK2 (Cao et al., 2009). This drastic change in circadian rhythm may cause various diseases ranging from socio-psychological diseases (Wulff et al., 2010) and metabolic syndrome (Maury et al., 2010), to various types of cancer (Savvidis and Koutsilieris, 2012). Excess phosphorylation of CK2 with PER protein switches the pathological state to rhythmic death pattern which could be apoptosis signature.

We then calculated permutation entropy ( $H_{P_{N}})$ of the time series of $P_{N}$ as a function of $k_{31}$ for individual interactions of CK2 with $P_{0},P_{1},\mathrm{and}\;P_{2}$ (Figure 4C). We found that the three circadian states can be detected distinctly for each time series, as in the previous simulation, and $H_{P_{N}}$ can be used as a parameter to detect these states which can be useful to clinicians and medical practitioners (Figure 4D).

**Figure 5:**
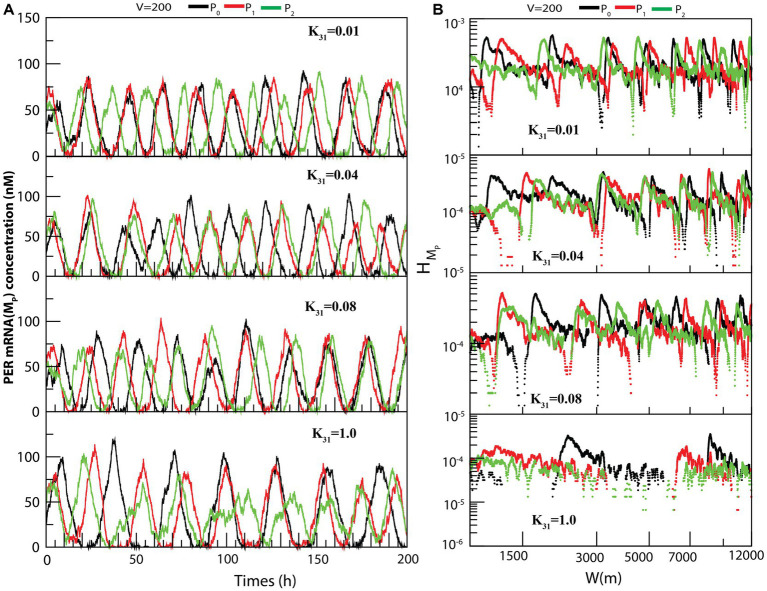
The dynamical behavior of $M_{P}$ on different configurational interaction of CK2 with PER gene mutants: **(A)** Dynamics of PER mRNA ( $M_{P}$ ) for different values of CK2 synthesis rate ( $k_{31}$ ) when CK2 interacts with different PER protein ( $P_{0}$ , $P_{1}$ and $P_{2}$ ). **(B)** Corresponding permutation entropy spectra as a function of W (m) for the corresponding time series on $M_{P}$ .

The dynamics of *MP* (Figure 5A) exhibited similarity to that of $P_{N}\;$ (as seen in Figure 4A). We also measured the permutation entropies of *MP* at fixed CK2 concentration and variation in system size. The observations from permutation entropies of *MP* at the three dynamical states of *MP* (Figure 5B) were similar to those of $P_{N}$ .

### Noise can regulate the circadian states

**Figure 6:**
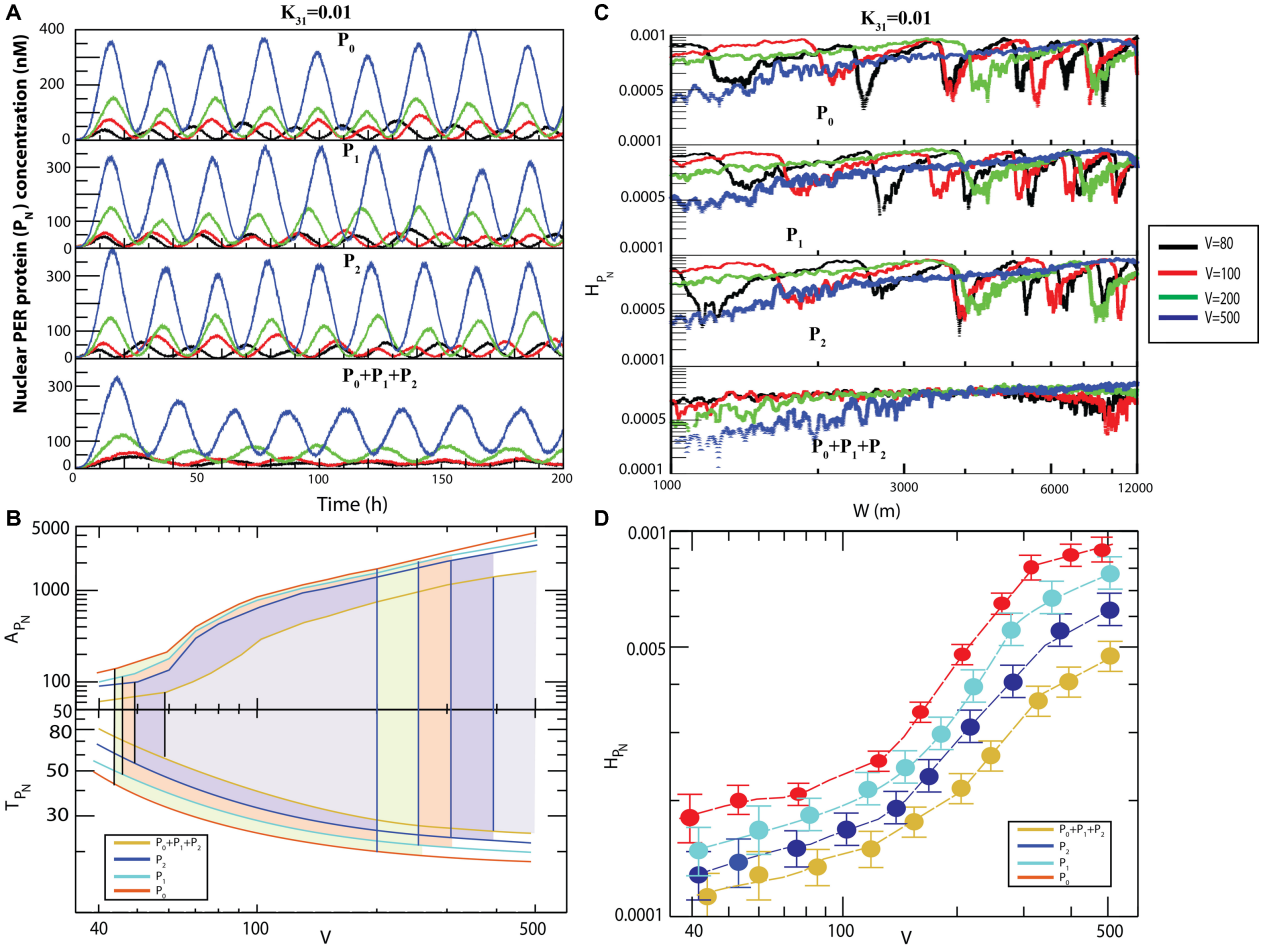
Noise-induced $P_{N}$ dynamics for all possible configurational interactions of CK2 with $P_{0}$ , $P_{1}$ and $P_{2}$ . **(A)** Dynamics of $P_{N}$ for four different system sizes *V* = 80, 100, 200, and 500 for fixed value of $k_{31}$ = 0.01. **(B)** Plots of $A_{P_{N}}$ and $T_{P_{N}}$ as a function of V for all four configurational interactions of CK2 with $P_{0}$ , $P_{1}$ and $P_{2}$ keeping $k_{31}$ = 0.01. **(C)** Plots of $H_{P_{N}}$ with respect to W for the corresponding system size values. **(D)** Permutation entropy $H_{P_{N}}$ with respect to V for all four possible configurational interaction of CK2 with $P_{0}$ , $P_{1},$ $P_{2}\;and\;P_{0}$ , $+P_{1}$ + *P*_2_.

Noise is an inherent property associated with the dynamics of any natural system (Glass, 2001; Bhadana et al., 2019, 2021). We studied the dynamics of $P_{N}$ for four possible configurational interactions of CK2 with the cytosolic PER proteins $(P_{1},$ $P_{2},and\;\left[P_{0}+P_{1}+P_{2}\right]),$ as we did in the previous simulation by keeping $k_{31}$ fixed at $k_{31}=0.01$ (which is associated with regular pattern of active circadian rhythm), and changing the strength of the noise $\xi \propto \frac{1}{\sqrt{V}}\;$ with four values for V as 80,100,200, and 500 (Figure 6). In all the configurational interactions of CK2 with the PER protein, we observed that an increase in noise (i.e., decrease in the value of V ) can alter the $P_{N}$ dynamics in each of the three circadian states. This indicates that noise can regulate the dynamics of circadian rhythm.

Generally, the size of an animal cell (of both the proliferation and non-proliferation types) can change due to various reasons (Lloyd, 2013), such as cell cycle progression and cell growth (Conlon and Raff, 2003), pathological states in muscle cells and starvation (Bodine et al., 2001), tumor or cancer progression (Lum et al., 2005), defects in synaptic wiring/rewiring in neurons (Crino, 2011), and manipulation of extracellular signals to prevent apoptosis (Lum et al., 2005). Animal cells can have size variability of up to ~59% of the normal cell size (Lum et al., 2005), and this change in the cell size can drastically affect molecular crowding within the cell (Feig et al., 2017). This variation in the system’s size is reflected in the dynamics of the system’s variables as internal noise fluctuation ( $\xi \propto \frac{1}{\sqrt{V}}\;$ ) (Gillespie, 2000). Since the circadian rhythm system is governed by well-defined reaction channels (Table 2), this change in molecular crowding could cause two significant impacts on the system. Firstly, it allows changes in the rate of interaction of the clock proteins in the system. This change in molecular interaction leads to changes in the internal noise associated with the system reflected in the dynamics of the constituting variables $P_{N},M_{P,}$ and of other clock proteins in the system. Hence, excess noise may destroy the signal associated with the system variables and may lead to external collapse of the system itself. Secondly, this molecular crowding may trigger changes in molecular traffic in the system; traffic jam due to excess molecular crowding may make the system unable to perform normal functions. In such a scenario, the amplitude is minimized, and the system faces death with infinitely large time period (Figure 6). Similar behavior can be observed in the other three configurational interaction models of CK2 with each of $P_{0},P_{1,}$ and $P_{2},$ particularly, in the interaction of CK2 with $P_{2}$ wherein the noise was slightly more sensitive (Figure 6A).

Results of calculations to derive the amplitudes $A_{P_{N}}$ and time periods $T_{P_{N}}$ of $P_{N}$ dynamics as a function of V for four different configurational interaction of CK2 with $P_{0},P_{1,}$ and $P_{2}$ are presented in Figure 6B. The results indicated that noise, measured by V, could drive the system to the three distinct states, namely active, weak activity, and rhythmic death. Hence, it can be inferred that noise is an important parameter that can trigger the system at various rhythmic states and can regulate the dynamics of the system. Permutation entropy spectra of $P_{N}$ corresponding to all the four models as a function of V values are presented in Figure 6C. We delineated the measure of complexity in three different derived states driven by CK2 by calculating permutation entropy $H_{P_{N}}$ of the dynamics of $P_{N}$ for all possible configurational interaction of CK2 with cytosolic PER proteins ( $P_{0},P_{1}$ , $P_{2,}$ and $P_{0}+P_{1}+P_{2})$ . Further, it can be observed that the measure of $H_{P_{N}}$ as a function of V can distinctly classify the three different circadian rhythm states (Figure 6C). Upon denoting $H_{P_{N},}^{A}\;H_{P_{N}}^{WA},$ and $H_{P_{N}}^{RD}$ as permutation entropies corresponding to the circadian states of active, weak activity, and rhythmic death, it can be inferred from the results that $H_{P_{N}}^{A}>H_{P_{N}}^{WA}>H_{P_{N}}^{RD}$ (Figure 6D).

**Figure 7:**
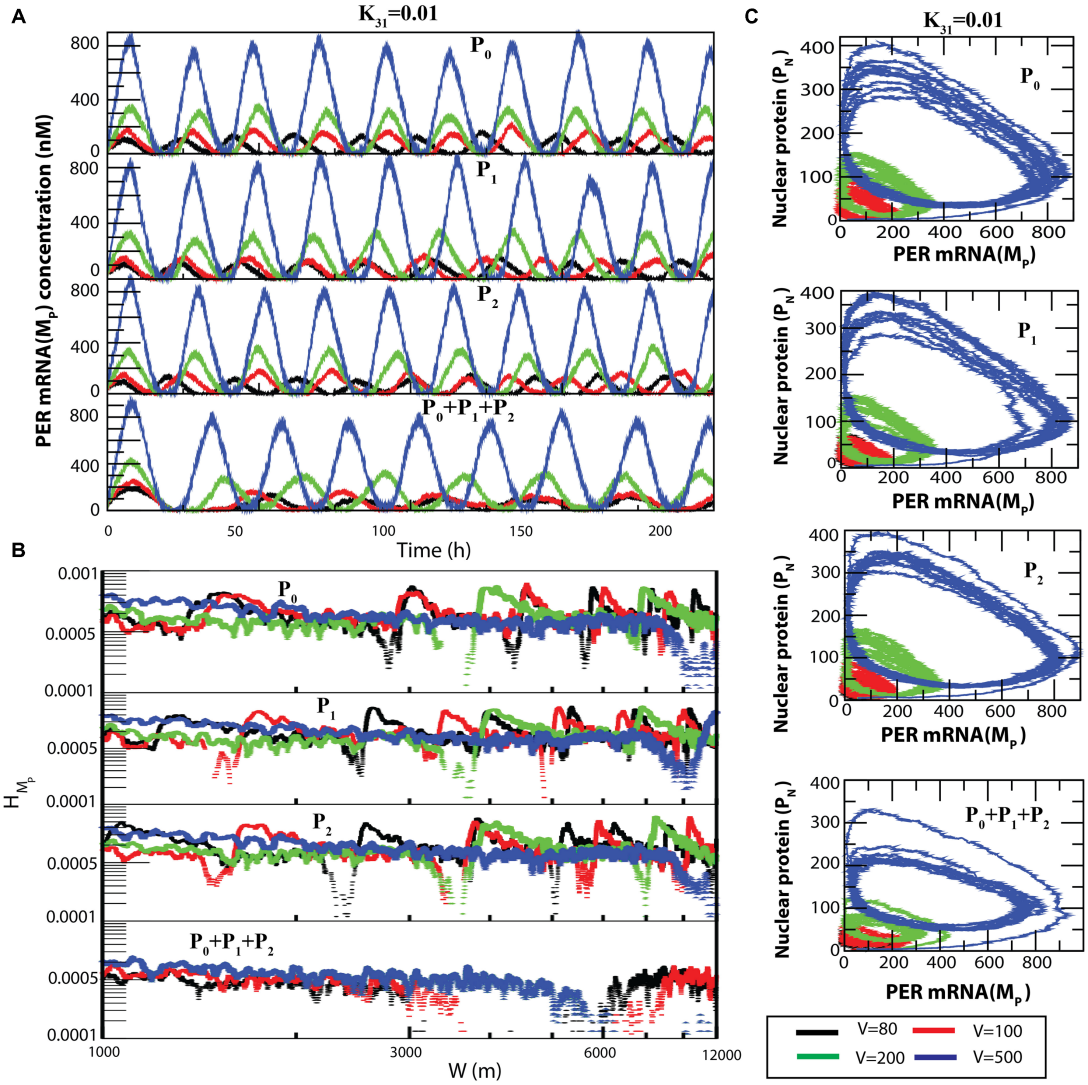
Noise-induced $M_{P}$ dynamics for all possible configurational interactions of CK2 with P0, P1 and P2. **(A)** Dynamics of $M_{P}$ for four different system sizes *V* = 80, 100, 200, and 500 for fixed value of $k_{31}$ = 0.01. **(B)** Plots of $H_{M_{P}}$ with respect to W for the corresponding system size values. **(C)** The two-dimensional plots of $P_{N}\;$ and $M_{P}$ for the four corresponding *V* values.

A similar behavior can be seen in the case of $M_{P}\;$ (Figure 7A) and $H_{\mathrm{MP}}$ (Figure 7B) dynamics. The transition from sustained oscillation fluctuations (active state) to amplitude death (rhythmic death) *via* the weak activity state can be clearly seen in the two-dimensional plot ( $P_{N}$ , $M_{P}$ ) as presented in Figure 7C.

### Circadian per-induced cellular pathways that can trigger pathological states

**Figure 8:**
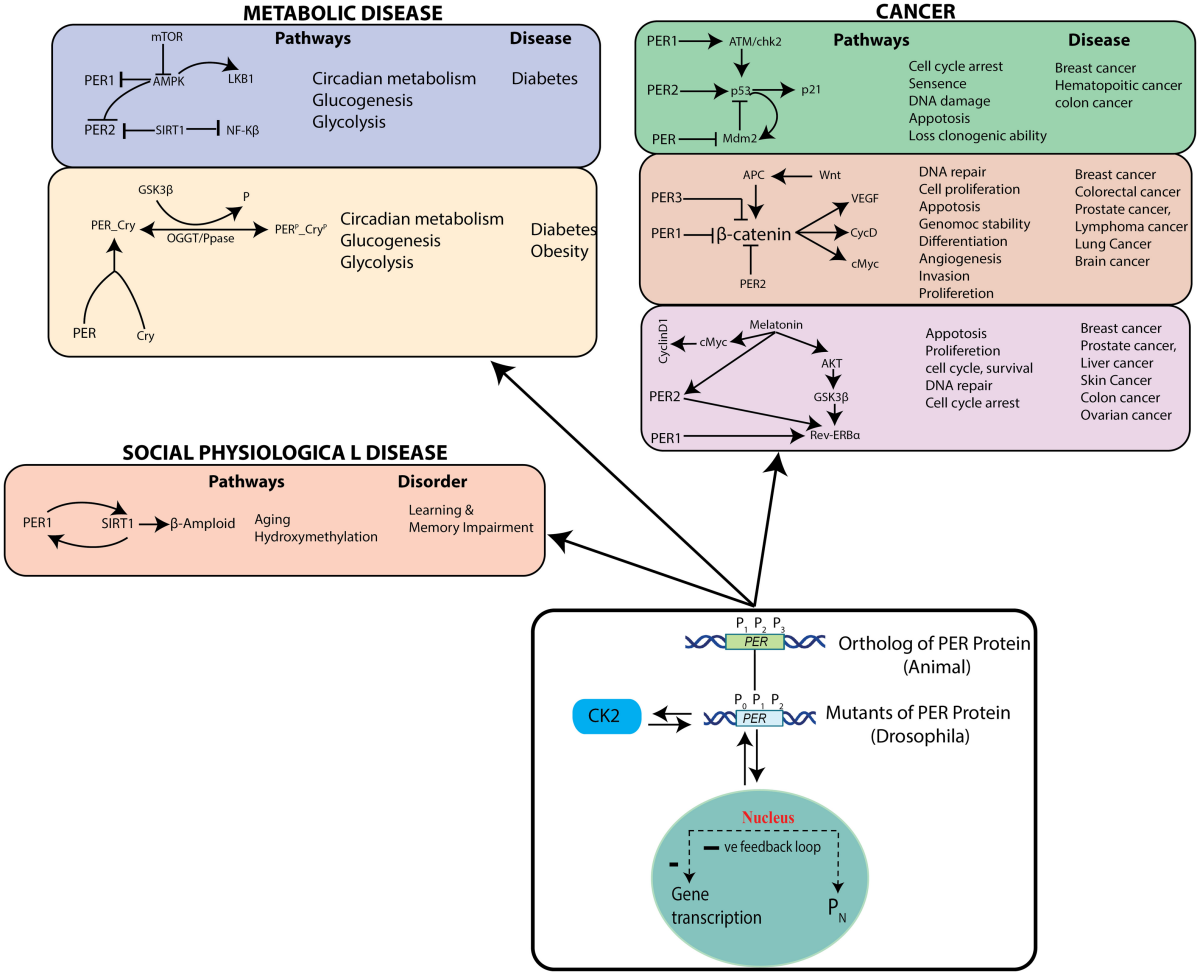
Possible pathological pathways driven by PER gene variations – List of the pathological pathways which can be affected by variations in the rhythms of PER gene mutants, and possible diseases.

We then identified cellular pathways that could be triggered by *PER* mutants (P_0_, P_1,_ and P_2_) in the circadian rhythm model (Figure 8). These cellular pathways can be grouped into three categories, cancer pathways (Savvidis and Koutsilieris, 2012; Papagiannakopoulos et al., 2016; Lee et al., 2019), socio–psychological pathways (Foster et al., 2013), and metabolic pathways (Maury et al., 2010). *PER* genes with their own rhythms generally regulate expressions of other genes participating in various important cellular pathways, significant changes of which may lead to various diseases. Since disruptions in the dynamics of $P_{0},P_{1},P_{2},$ and $P_{N}$ in circadian rhythm may lead to drastic changes in the dynamics and mechanisms of these closely interacting pathways and to various pathological states, they can cause various diseases. Dysfunctional PER proteins disturb key biological functions, such as cell proliferation, DNA damage, cell cycle, and apoptosis, resulting in various type of cancers (Deibel et al., 2015).

According to various studies, there may exist a tissue-dependent relationship between insulin resistance (or type 2 diabetes, T2D) and dysregulated molecular clock activity (Hart, 2013; Gabriel et al., 2021). In a time-course experiment (Jakubowicz et al., 2017) with white adipose tissue biopsies from people with healthy weight or obesity or T2D, no variation in the rhythm and amplitude of core-clock (PER1, PER2, PER3, DBP, BMAL1, and CRY2), metabolic (PGC1), and clock-related (REVERB) genes could be observed in biopsy tissues (Jakubowicz et al., 2017). On the other hand, when the sleep–wake cycle and dietary factors were controlled, it was seen that the amplitude oscillations of core-clock genes and a number of rhythmic genes are decreased in adipose tissues from patients with T2D, as compared to lean and healthy individuals (Stenvers et al., 2019). The mRNA expression of BMAL1, PER1, PER2, and PER3 in leukocytes was observed to be lower in non-diabetic individuals, as compared to those with diabetes (Hart, 2013; Stenvers et al., 2019). Additionally, the expression levels of PER1, PER3, and BMAL1 molecular-clock genes in leukocytes obtained from patients with T2D was seen inversely correlated with hemoglobin A1C (HbA1c) levels, indicating an association between insulin resistance and T2D (Gabriel et al., 2021). Expression of PER2, PER3, and CRY2 mRNA were also significantly associated with plasma HbA1c levels and islet insulin content in pancreatic islets from healthy people and from those with T2D (Gabriel et al., 2021). Uncertainties still exist regarding the fundamental mechanisms that regulate metabolic rhythmicity, especially regarding whether rhythmicity is lost in T2D. Mutations in clock genes were initially associated with glucose homeostasis (Rudic et al., 2004) and later with hyperinsulinemia, hyperglycaemia, and obesity in murine models (Turek et al., 2005). Numerous single nucleotide polymorphisms (SNPs) in human clock genes, including rs18012602, rs4580704, rs4864584, rs3749474 and rs1464490, have also been associated with obesity (Sookoian et al., 2008), hyperglycaemia, and a higher prevalence of T2D (Sookoian et al., 2008).

Aberrations in the PER protein disrupts multiple biological functions, such as age-related hydroxy methylation, which causes increased risk for socio–psychological diseases (e.g., learning and memory impairment, and Alzheimer’s disease) (Liu and Chang, 2017). Significant changes in the dynamics of $P_{0},P_{1},P_{2}$ and $P_{N}$ in the circadian rhythm and noise in the system may lead to drastic changes in the dynamics and mechanisms of pathways and to various pathological states, causing diseases corresponding to these pathways. Therefore, it is crucial to identify pathways that are significantly affected by changes in the $P_{0},P_{1},P_{2},$ and $P_{N}$ dynamics.

On the other hand, patients having any one or more of the diseases mentioned in Figure 8 can become healthy by maintaining a proper circadian rhythm (Foster et al., 2013), and the process could possibly be termed as a reversal of pathological state. However, even though this may be possible for some diseases, one needs to categorically study these possibilities from both the mathematical modeling and experimental points of view. In accordance with these postulations, previous reports have also recommended studies on genes/proteins in the circadian rhythm pathways as potential drug targets (Wood et al., 2009).

## Discussion

Circadian rhythm is one of the most important biological rhythms that can regulate and interfere with various biological processes, such as those involved in cell regeneration, hormone production, and controlling brain activity. In the present work, we studied the impact of CK2 and stochastic noise on a circadian rhythm model. It has been reported in literature that CK2 promotes the progressive phosphorylation of clock protein that leads to the rapid degradation of hyperphosphorylated isoforms by the ubiquitin–proteasome pathway. Our results suggest that the presence of CK2 in the system has a strong impact on its dynamics, as reflected in the time evolution of the nuclear PER protein and mRNA. We found that CK2 drives three distinct circadian rhythm states, namely active, weak activity, and rhythmic death. The active state corresponds to the time period of oscillation ( T ) in the rhythm variable ( $P_{N},M_{P}$ etc) as approximately 22–24 h, with optimal amplitude (*A*). When T is larger than that of the active state, it is termed as weak activity, whereas the circadian state corresponding to T→∞ , where A→0 is known as rhythmic death (Konopka and Benzer, 1971; van Soest et al., 2020).

Noise is an inherent property of any natural system. An interesting and important role of noise in a system is its capability to control the behavior of the system. The size of an animal cell can be changed due to various intracellular and extracellular factors (Lloyd, 2013); this variation in size is reflected in internal noise fluctuation in the system’s dynamics. In our study, we observed that noise can trigger the three circadian states and can control the behavior of the system. Our observations on the impact of noise (due to defects in biochemical reactions) on the circadian states support the hypothesis that the circadian clock is highly sensitive to CK2 activity.

Radical changes in circadian rhythm can occur due to various reasons, such as genetic defects, irregular work shifts, and aging (Jakubowicz et al., 2017). Change in the circadian state leading to weak activity may result in various diseases, especially cancer. This is because variations in the *PER* gene (denoted as $P_{0},P_{1},P_{2}$ in drosophila and as $P_{1},P_{2},P_{3}$ in human) may affect disruption in the cell cycle *via* the cMyc pathway with PER, ATK pathway with PER1 and estrogen signal, and HER with *PER* mutants; these pathways are known to be signature for progression of cancer in organs such as breast (Konopka and Benzer, 1971). Further, since binding of PER to androgen receptor (AR) causes the inhibition of AR transcriptional activity, the disruption of circadian rhythm may cause prostate cancer (Cao et al., 2009). Apart from prostate cancer, defects in circadian rhythm may cause various other cancer types (Savvidis and Koutsilieris, 2012), such as colorectal cancer due to the PER2–ATM–Chk1/Chk2 pathway (Mazzoccoli et al., 2011).

Disruptions in circadian rhythm may also cause several other disorders, such as psychiatric and neurodegenerative diseases (Wulff et al., 2010), jet lag disorder, and mental illness (Foster et al., 2013), and illnesses due to aging (Deibel et al., 2015). This could be because circadian rhythm is associated with various important cellular pathways. Some of the circadian genes/proteins are found to preserve cellular stability; for instance, PER1 is experimentally found to be anti-apoptotic in nature. Jet lag refers to misalignment of body’s internal clock with the local time at the destination. The jet lag phenomenon often occurs when flying across two or more time zones. The symptoms include sleeping problems, impaired thinking, hampered physical function and stomach problems. In rare instances, jet lag leads to sleep paralysis and seizures. Jet lag, in the case of people taking frequent long-haul flights, can be a long-term problem. Chronic circadian rhythm disruption can raise the risk of chronic disorders such as diabetes, depression and cancer (Foster et al., 2013). Hence, one must maintain proper circadian rhythm in their day-to-day life.

## Conclusion

We studied the model for circadian rhythm using stochastic simulation algorithm, and examined the behavior of the amplitude, time period and permutation entropy of PER proteins to identify three distinct circadian states, namely active, weak activity, and rhythmic death all driven by CK2 protein. The interaction between changes in the *PER* gene expression under certain conditions illustrate the need for mathematical models to understand the underlying processes. Although a full representation of biological systems is hard to achieve owing to modeling limitations, the present study might help to understand how complex oscillatory dynamics occur at the molecular level. However, experimental data are needed to validate such phenomena at molecular level of the circadian clock.

## Data availability statement

The original contributions presented in the study are included in the article/supplementary material, further inquiries can be directed to the corresponding authors.

## Author contributions

MZM, RKB, and TAT: conception and design. MZM, RKB, FA-M, and TAT: development of methodology and drafting of the manuscript. MZM, YF, MD, AC, RKB, FA-M, and TAT: analysis and interpretation of the data. MZM, RKB, MD, and TAT: statistical analysis. All authors read and approved the final manuscript.

## Conflict of interest

The authors declare that the research was conducted in the absence of any commercial or financial relationships that could be construed as a potential conflict of interest.

## Publisher’s note

All claims expressed in this article are solely those of the authors and do not necessarily represent those of their affiliated organizations, or those of the publisher, the editors and the reviewers. Any product that may be evaluated in this article, or claim that may be made by its manufacturer, is not guaranteed or endorsed by the publisher.
